# A method to improve quantitative radiotracing‐based analysis of the in vivo biodistribution of drug carriers

**DOI:** 10.1002/btm2.10208

**Published:** 2021-02-13

**Authors:** Nikša Roki, Melani Solomon, Lou Casta, Jessica Bowers, Robert C. Getts, Silvia Muro

**Affiliations:** ^1^ Fischell Department of Bioengineering University of Maryland College Park Maryland USA; ^2^ Institute for Bioscience and Biotechnology Research, University of Maryland College Park Maryland USA; ^3^ Genisphere, LLC Hatfield Pennsylvania USA; ^4^ Institute for Bioengineering of Catalonia of the Barcelona Institute of Science and Technology Barcelona Spain; ^5^ Institution of Catalonia for Research and Advanced Studies Barcelona Spain; ^6^ Present address: Code Biotherapeutics, Hatfield, Pennsylvania USA

**Keywords:** biodistribution data correction, degradation, drug delivery carriers, free label, in vivo biodistribution, radiotracing, trichloroacetic acid precipitation

## Abstract

Biodistribution studies are essential in drug carrier design and translation, and radiotracing provides a sensitive quantitation for this purpose. Yet, for biodegradable formulations, small amounts of free‐label signal may arise prior to or immediately after injection in animal models, causing potentially confounding biodistribution results. In this study, we refined a method to overcome this obstacle. First, we verified free signal generation in animal samples and then, mimicking it in a controllable setting, we injected mice intravenously with a radiolabeled drug carrier formulation (^125^I‐antibody/3DNA) containing a known amount of free radiolabel (^125^I), or free ^125^I alone as a control. Corrected biodistribution data were obtained by separating the free radiolabel from blood and organs postmortem, using trichloroacetic acid precipitation, and subtracting the confounding signal from each tissue measurement. Control free ^125^I‐radiolabel was detected at ≥85% accuracy in blood and tissues, validating the method. It biodistributed very heterogeneously among organs (0.6–39 %ID/g), indicating that any free ^125^I generated in the body or present in an injected formulation cannot be simply corrected to the free‐label fraction in the original preparation, but the free label must be empirically measured in each organ. Application of this method to the biodistribution of ^125^I‐antibody/3DNA, including formulations directed to endothelial target ICAM‐1, showed accurate classification of free ^125^I species in blood and tissues. In addition, this technique rendered data on the in vivo degradation of the traced agents over time. Thus, this is a valuable technique to obtain accurate measurements of biodistribution using ^125^I and possibly other radiotracers.

## Graphical abstract

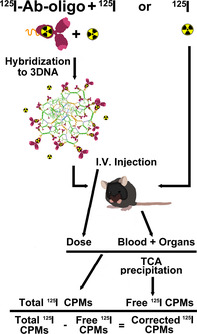

## 1 INTRODUCTION

Drug delivery systems (DDSs) can be used to improve a drug's therapeutic index by adapting its bio‐physicochemical properties to the requirements of pathological conditions.^1^, ^2^, ^3^, ^4^ An important strategy is active targeting, by which a therapeutic agent can preferentially accumulate at the sites of interest, lowering side effects.^3^ Therefore, the biodistribution of DDSs in an organism provides valuable information on the possible efficacy and side effects of the drug.^5^ As such, biodistribution studies are essential in guiding DDS design and preclinical testing.^5^, ^6^

There are many methods to measure biodistribution, including (i) noninvasive in vivo strategies,^6^, ^7^, ^8^, ^9^, ^10^, ^11^, ^12^, ^13^ such as magnetic resonance imaging (MRI), positron generating dual γ emission tomography (PET), single γ photon emission computed tomography (SPECT), optical fluorescence or bioluminescence imaging, and X‐ray computed tomography (CT); and (ii) highly sensitive ex vivo approaches,^6^, ^14^, ^15^, ^16^, ^17^, ^18^, ^19^, ^20^, ^21^, ^22^ including radioisotope tracing (radiotracing), spectrometry and spectroscopy, and high‐pressure liquid chromatography.

Radiotracing methods are interesting because they provide high sensitivity.^6^, ^7^ They require labeling agents with a radioisotope or substitutive incorporation during synthesis.^23^, ^24^ Agents of known specific activity are administered in vivo, followed by collection of tissues for ex vivo measurements. As an example, ^125^I can be used to label peptides, proteins, nucleic acids, nanoscale materials, and so on, enabling their quantification in a γ‐counter.^6^ While the inherent risks of using γ emitters must be balanced against the convenience of analysis, ex vivo γ‐radiotracing provides standardized quantitation without tissue matrix interference, complex processing, routine calibrations, standard curves or quality control samples, while providing sub‐parts‐per‐billion sensitivity and multiplexing capacity.^6^, ^23^, ^24^ Signal loss due to radioactive decay is known for each radioisotope and can be quantitated. Numerous publications validate the use of ex vivo γ radiotracing for biodistribution studies of drugs and their carriers.^25^, ^26^, ^27^, ^28^, ^29^, ^30^

Yet, like other methods, radiotracing may suffer from a noise signal that originates from the presence of free label. Free label, for example, radio‐iodine and other radiohalogens, may arise by detachment from DDSs upon interaction with the body or degradation of biodegradable DDS components. Polymers, targeting antibodies, and/or cargoes could be labeled with a radioisotope and their body degradation could release their monomolecular building blocks linked to the respective radiolabel (herein called “free label” for simplicity). Free label could also be released prior to administration, depending on the DDS shelf‐life. Due to the high sensitivity of radiotracing, free label can confound biodistribution results due to their different accumulation profile versus a DDS. Since this cannot be predicted, organ‐specific corrections of the measurements are essential. Modification of the radiotracing biodistribution method is necessary such that the true signal originating from the label attached to the DDS formulation can be distinguished from the signal originating from any possible free label.

Here, we investigated whether precipitation with trichloroacetic acid (TCA), a method used in vitro to separate free radiolabel from radiolabeled biological polymers, such as proteins or nucleic acids,^31^, ^32^ could be applied to precisely elucidate the in vivo biodistribution of a DDS. Results demonstrate that small amounts of free label do generate in the biological milieu and that this method can be used to identify free ^125^I present in blood and organs, providing a valuable means for correction of free radiolabel present in samples that are used for biodistribution studies.

## 2 RESULTS

### 2.1 In vitro generation of free ^125^I label from ^125^I‐Ab/3DNA in conditions mimicking pre‐ and post‐in vivo administration

Small amounts of tracer may arise from radiolabeled DDS, which could confound biodistribution studies. To illustrate this, we used a DDS called 3DNA®, a DNA construct assembled from single‐stranded polynucleotides designed to hybridize into modules and layers, via sequence complementarity^33^ (section 4), resulting in a drug nanocarrier.^33^, ^34^, ^35^, ^36^ The 3DNA formulation used here has been recently characterized^37^ and has 170 ± 7 nm hydrodynamic diameter, −19 ± 0.6 mV ζ‐potential, and 0.22 ± 0.003 polydispersity index (PDI). To mimic formulations used in targeting strategies, these nanocarriers were coupled to an antibody (Ab) labeled with ^125^I (^125^I‐Ab) and modified with an oligonucleotide complementary to 3DNA outer arms,^35^, ^37^, ^38^ which increased the average hydrodynamic diameter to 181 ± 5 nm and decreased the ζ‐potential to −42 ± 0.5 mV, with similar PDI of 0.23 ± 0.019.

**FIGURE 1:**
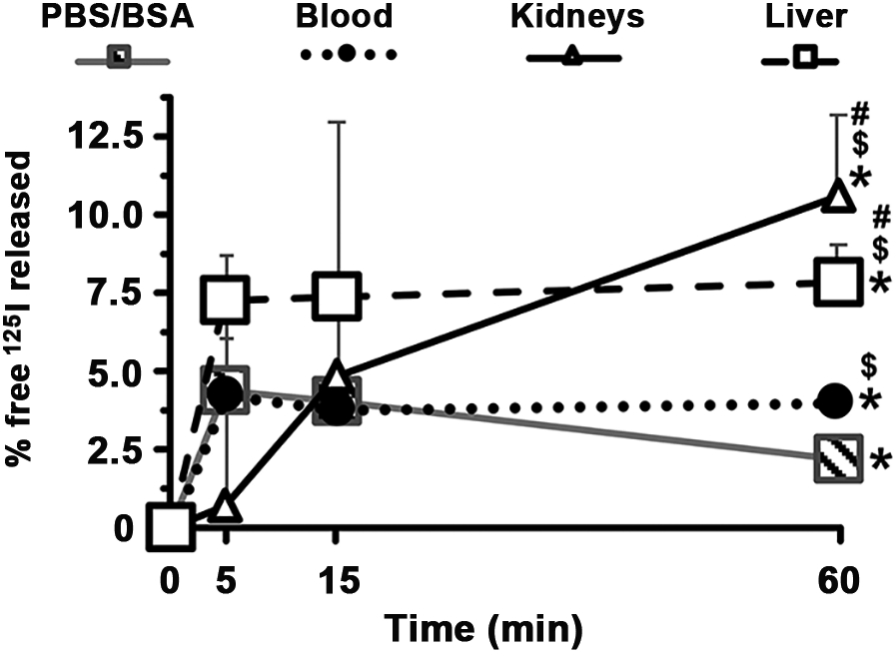
In vitro generation of free ^125^I from ^125^I‐Ab/3DNA in pre‐ and post‐administration conditions. ^125^I‐Ab/3DNA was incubated with either control buffer (3% BSA in PBS) or tissue samples (blood and homogenized kidney or liver) from C57BL/6 mice. Percentage of free ^125^I was calculated from the CPM corresponding to the total ^125^I versus free ^125^I in each sample as determined from radioactivity measurements in γ‐counter prior and after trichloroacetic acid (TCA) precipitation, respectively. Data are mean ± S.D. (*n* = 3–4). *Compares 60 min versus 0 min, for each condition; $ compares blood, kidneys, and liver versus PBS/BSA, # compares kidneys and liver versus blood condition; (*p* < 0.05 by Student's *t*‐test)

We tested the possible release of free ^125^I from ^125^I‐Ab/3DNA (Figure 1) by incubating this formulation with injection buffer (pre‐administration simulation), mouse blood, liver or kidney homogenates (post‐administration simulation). Free ^125^I was separated by adapting an established method that employs TCA, an agent used to precipitate biological polymers (proteins, nucleic acids, and polysaccharides to some extent), allowing easy separation of free label after centrifugation.^31^, ^32^ Release of small amounts of free ^125^I was detected in pre‐ and post‐ administration simulations, ranging from 2% to 11% (at 60 min) of the total signal. Most of the label release occurred in the first 5 min (4, 4, 7% release for buffer, blood, and liver), indicating a fast event. Free‐label release was similar in buffer and blood for early time points, and increased in blood by 60 min, as expected. Also as expected, release was greater in the liver and kidney homogenates versus the blood and buffer. This highlights that: (a) there is low (≤11) release of free label from ^125^I‐Ab/3DNA in physiological conditions, validating that this radiolabel is sufficiently stable to trace biodistribution within the selected time range; and (b) there is some degree of fast label release pre‐ and post‐administration (4% in buffer and blood at 5 min). Thus, there is a need to trace this confounding signal, for which we compared the biodistribution upon i.v. administration in mice of ^125^I‐Ab/3DNA containing known amounts of free ^125^I (a controlled situation) versus free ^125^I alone, measured prior to and after TCA precipitation (Figure S1).

### 2.2 Biodistribution of i.v. administered free ^125^I radiolabel

**FIGURE 2:**
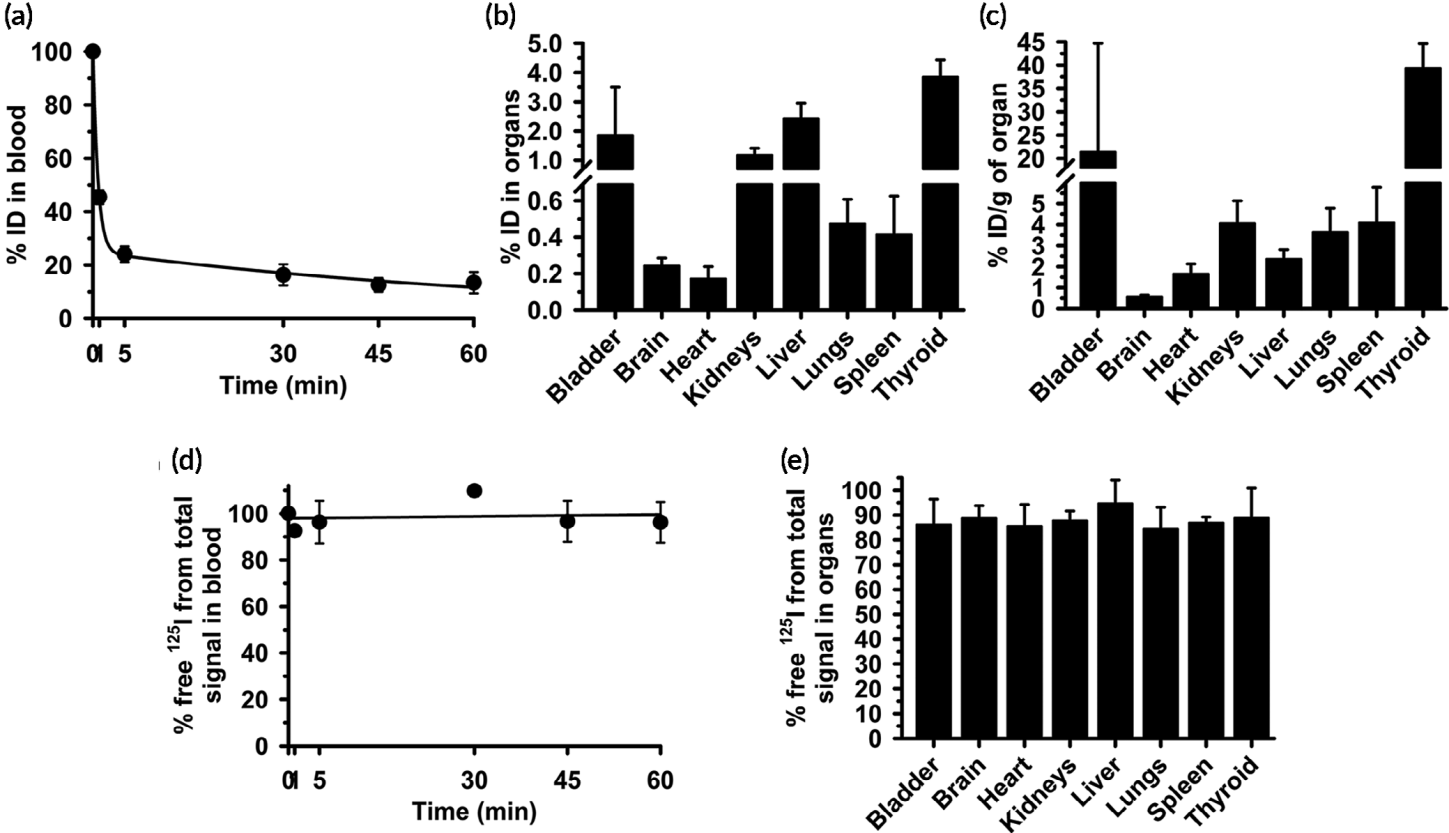
Biodistribution of control free ^125^I. Free ^125^I control was i.v. injected in C57BL/6 mice. (a) Blood samples were collected at the indicated post‐injection times and (b, c) organs were obtained at sacrifice at 60 min, weighed and measured in γ‐counter to calculate the percentage of the injected dose (%ID) found in (a) blood and (b) organs. (c) %ID per gram organ (%ID/g) was calculated to compare the relative “concentrations” of free ^125^I in organs, given their very different weight. (d) Percentage of the total CPM detected in blood or (e) tissue sample, which were identified as free ^125^I after TCA precipitation. Data are mean ± S.D. (*n* ≥ 5 mice)

We determined the biodistribution of free ^125^I injected i.v. in mice. As expected for such a small molecule, we observed fast blood clearance (Figure 2(a)): only 46% and 24% of the injected dose (%ID) remained in blood at 1 min and 5 min. This was followed by slow clearance until 1 h: 16 %ID and 13 %ID were found at 30 min and 60 min (Figure 2(a)). The half‐life (t_1/2_), area under the curve (AUC), clearance, and mean residence time (MRT) were 1.3 min, 178.3 %ID×min, 0.6 %ID/min, and 1.9 min, respectively (Table S1). Free ^125^I accumulated in small quantities in organs (Figure 2(b)): the sum of all radioactivity measured in the bladder, brain, heart, kidneys, liver, lungs, spleen, and thyroid gland was 11 %ID, suggesting that free ^125^I may be quickly excreted. Indeed, free ^125^I was predominantly found in the bladder, kidneys and liver (2, 1, and 2 %ID; Figure 2(b)), and the thyroid (4 %ID; Figure 2(b)).

To estimate the free ^125^I “concentration” in each site, we calculated the %ID per gram of tissue (%ID/g; Figure 2(c)). The highest concentrations were found in the thyroid and bladder (39 and 21 % ID/g; Figure 2(c)), as expected. The liver concentration was only 2 %ID/g, indicating that accumulation in this organ is mostly due to liver's large size and open vasculature rather than specificity. The brain, heart, kidneys, lungs, and spleen also had low free ^125^I amounts and concentrations (Figure 2(b,c)).

Then, we aimed to verify that this label can be detected as free ^125^I in tissue samples. The organs from the described experiment were homogenized and mixed with TCA, while blood samples were directly mixed with this reagent. After precipitation and centrifugation, an aliquot of the supernatant was assessed for free ^125^I. This demonstrated that all ^125^I present in the body was detected as a free species: 100% and ≥84% of all ^125^I in blood (Figure 2(d)) and organs (Figure 2(e)), respectively. This was independent of the absolute level or concentration of ^125^I found: 100% of all ^125^I detected in blood was in free form although at early and late time points the biodistribution of the total radiolabel was quite different (46 %ID at 1 min vs. 13 %ID at 60 min); similarly, organs with higher or lower ^125^I concentrations (39 %ID/g in the thyroid vs. 0.6% ID/g in the brain) contained ~90% free ^125^I. Thus, the method employed enables accurate detection of any free radiolabel present and may be used to correct total CPM data for the free radiolabel fraction found in blood and organs.

### 2.3 Implementation of control, free ^125^I corrections on the biodistribution of ^125^I‐Ab/3DNA

Free ^125^I biodistribution ranged broadly among different organs, that is, from 0.6 %ID/g in the brain to 39 %ID/g in the thyroid (Figure 2(c)). This indicates that the presence of free label in a DDS formulation needs to be corrected by its own free ^125^I biodistribution and, as such, the %ID observed in each organ (Figure 2) could be used to correct carrier biodistribution data. Using the thyroid example, TCA implementation on the formulation to be injected would quantify the pre‐administration free ^125^I label, 4% of which should distribute to the thyroid per Figure 2(b). The free ^125^I CPM in this gland must be subtracted from the total CPM empirically measured for this tissue to determine the carriers found in the gland without any free ^125^I label.

**FIGURE 3:**
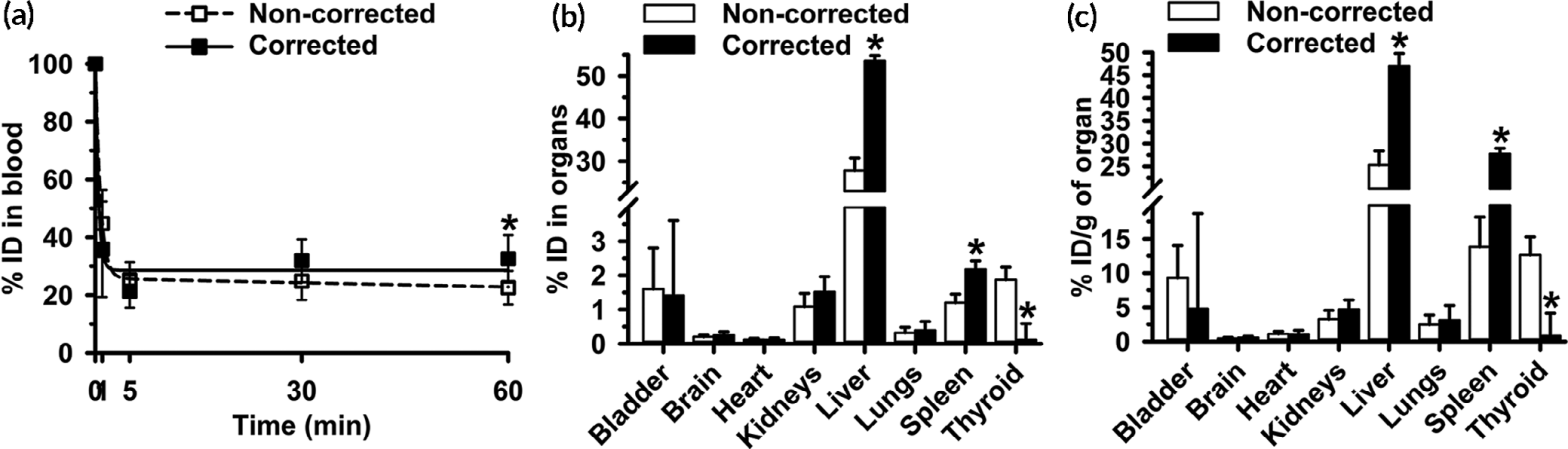
Control free ^125^I corrections of the ^125^I‐Ab/3DNA biodistribution. ^125^I‐Ab/3DNA was i.v. injected in C57BL/6 mice along with a known amount of free ^125^I. Blood and organs were collected and weighed at the indicated times and measured in a γ‐counter. Results were calculated from the total CPM measured in blood and organs and referred to as the total CPM of the injected dose (including ^125^I‐Ab/3DNA + free ^125^I for noncorrected data), or they were corrected to subtract in each tissue the expected biodistribution of free ^125^I obtained in Figure 2(a,b). (a) Circulation and (b) biodistribution are expressed as percentage of the injected dose (% ID). (c) %ID per gram organ (%ID/g) shows organ concentration. Data are mean ± S.D. (*n* ≥ 5 mice). *Compares corrected to noncorrected data (*p* < 0.05 by Student's *t*‐test)

To test this, we injected mice with ^125^I‐Ab/3DNA as previously,^35^, ^37^, ^38^ yet co‐administered with a known amount (40–50%) of free ^125^I label. We used a nonspecific Ab‐oligo to avoid biodistribution changes and cellular uptake driven by specific targeting, which may complicate interpretation. Validating this Ab/3DNA model, our publication^37^ shows that coupling of 3DNA to a specific Ab‐oligo recognizing intercellular adhesion molecule 1 (ICAM‐1), a protein preferentially expressed on the pulmonary endothelium, markedly increased lung‐specific targeting compared to controls.^37^ The circulation of the ^125^I‐Ab/3DNA + ^125^I formulation (herein called ^125^I‐Ab/3DNA for simplicity) was assessed at various times after administration and organ biodistribution was determined at 60 min (Figure 3). The total radiolabel disappeared quickly from the circulation (45 and 25 %ID at 1 min and 5 min; Figure 3(a) white symbols), as expected for a non‐PEGylated nanocarrier.^3^, ^30^ The t_1/2_, AUC, clearance, and MRT were 20.3 min, 1613.4 %ID×min, 0.1 %ID/min, and 29.3 min (Table S1). Yet, contrary to free ^125^I alone, ^125^I‐Ab/3DNA did not accumulate preferentially in the thyroid (2 %ID and 13 %ID/g; Figure 3(b,c) white bars) but in the liver (28 %ID and 25 %ID/g; Figure 3(b,c) white bars), as expected for a nanocarrier with an Ab coat.^3^, ^37^ These results describe the noncorrected data, but then, we implemented the explained correction (Figure 3, black symbols and bars). Corrected data showed some changes for the circulation of ^125^I‐Ab/3DNA (44% increase at 60 min; 1.4‐fold increase in AUC, Table S1) but, mainly, it lowered accumulation in the thyroid (by 94% for both the %ID and %ID/g of organ) and increased liver and spleen accumulation (by 92% and 82%, looking at the %ID parameter), expected changes for a nanosized formulation versus free ^125^I.

**FIGURE 4:**
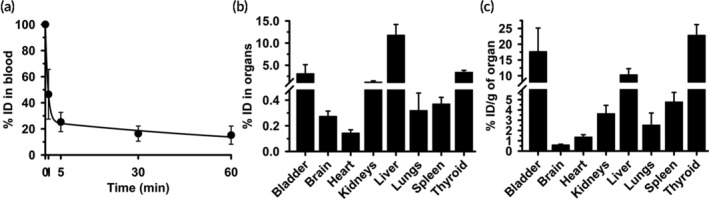
Biodistribution of free ^125^I contained in ^125^I‐Ab/3DNA model formulation. ^125^I‐Ab/3DNA containing a known amount of free ^125^I label was injected i.v. in C57BL/6 mice. (a) Blood samples were collected at the indicated post‐injection times and (b, c) organs were obtained at sacrifice at 60 min. TCA precipitation rendered CPM of free ^125^I in each blood and organ sample, from which the (b) percentage of the injected dose (%ID) was calculated by comparison to the total free ^125^I injected. (c) Similar data but referred as %ID per gram organ (%ID/g) to show organ concentration. Data are mean ± S.D. (*n* ≥ 5 mice)

To validate this correction, we performed TCA precipitation on the samples, to obtain the empirical level of free ^125^I in each of them. The level of free ^125^I measured in blood was ≈15 %ID at 60 min (Figure 4(a)), similar to that of free ^125^I injected alone (Figure 2(a)). The t_1/2_, AUC, and MRT were 1.5 min, 203.6 ID%×min, and 2.2 min (Table S1), also similar to the free ^125^I injection. The biodistribution varied broadly among organs (0.1 %ID in the heart and 3 %ID in the thyroid gland; Figure 4(b)), similar to free ^125^I variability (Figure 2(b)). However, unexpectedly, the biodistribution of free ^125^I within the ^125^I‐Ab/3DNA injection differed from that of free ^125^I control: %ID/g in kidneys decreased by 10%, both %ID and %ID/g in the lung decreased by 32% and 31%, %ID/g was reduced by 18% in the bladder and 42% in the thyroid (Figure 4(b,c)). Conversely, the %ID/g increased 17% for the spleen and both the %ID and %ID/g increased by 386% and 335% for the liver. Therefore, this correction that assumes a similar biodistribution of free ^125^I within the ^125^I‐Ab/3DNA as for the free ^125^I injected alone is not accurate.

Additional controls were performed to demonstrate if free ^125^I would interact with hemoglobin or albumin, as reported.^39^, ^40^ We incubated ^125^I‐Ab‐oligo (the radiolabeled counterpart in ^125^I‐Ab/3DNA) containing 10% free ^125^I with heparinized mouse blood or plasma. After 30 min, we performed TCA precipitation and found 11.8 ± 0.7% and 12.2 ± 0.75% free ^125^I in these mixtures. Independently, free ^125^I alone (100%) was incubated with whole blood or plasma, after which TCA precipitation rendered detection of 102.7 ± 2.0% and 110.8 ± 0.5% free ^125^I. Mixing of nonlabeled (cold) Ab/3DNA with free ^125^I followed by TCA precipitation, rendered 105.1 ± 0.4% free ^125^I detection, demonstrating that all the free label is detected despite the presence of the carrier. Mixing of cold Ab/3DNA with ^125^I‐Ab‐oligo containing 10% free ^125^I lead to detection of 11.0 ± 0.4% free iodine, demonstrating that even if some ^125^I‐Ab‐oligo counterpart were to detach from the 3DNA, this would not interfere with the ability to detect free ^125^I.

### 2.4 Implementation of empirical free ^125^I corrections on the biodistribution of ^125^I‐Ab/3DNA

**FIGURE 5:**
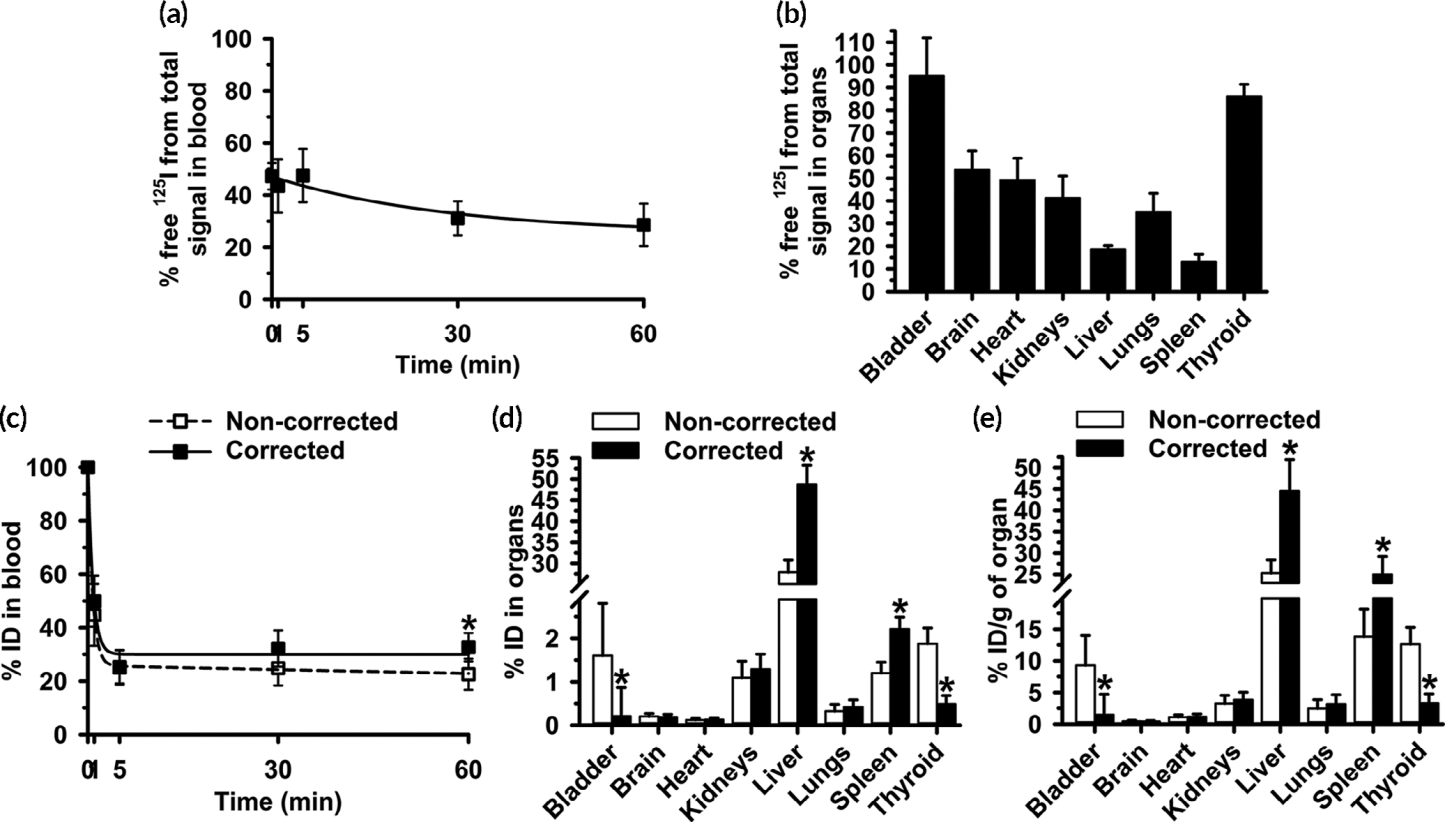
Contribution of free ^125^I to the total ^125^I signal found in blood and organs after ^125^I‐Ab/3DNA injection and respective biodistribution correction. C57BL/6 mice were injected with ^125^I‐Ab/3DNA containing a known amount of CPM of free ^125^I. (a) Blood samples were collected at the indicated times post injection and (b) organs were collected at sacrifice at 60 min for CPM measurements in γ‐counter. In both cases, total CPM and free ^125^I CPM were measured prior to and after TCA precipitation, so that the fraction of free ^125^I over the total CPM in each body compartment was calculated. (c) Circulation and (d) biodistribution of ^125^I‐Ab/3DNA expressed as percentage of the injected dose (% ID), which were calculated from the total CPM measured and referred to the total CPM of the injected dose (noncorrected), or corrected to subtract free ^125^I CPM in each tissue as well as the dose. (e) Similar data but referred as %ID per gram organ (%ID/g) to show organ concentration. Data are mean ± S.D. (*n* ≥ 5 mice). *Compares corrected to noncorrected data (*p* < 0.05 by Student's *t*‐test)

Therefore, free ^125^I in blood and organ samples must be empirically measured within each experiment, to subtract this value from the total CPM in said samples. To prove this, out of the total CPM found in each blood and organ sample, we calculated which fraction corresponded to free ^125^I, not true ^125^I‐Ab/3DNA (Figure 5). Results show that much of the label found in blood was free ^125^I (e.g., 29% of all CPM in this organ, at 60 min; Figure 5(a)), while in tissues this fraction varied broadly from 13% of all CPM in the spleen to 54% all CPM in the brain or 95% of all CPM in the bladder (Figure 5(b)).

The contribution of free ^125^I to the total CPM of each tissue sample was highly variable, validating that the best possible correction for “clean” carrier biodistribution data is to subtract free ^125^I counts in each organ from the total counts in said organ. Implementing this correction showed that the blood clearance profile remained similar, although a bit enhanced for the 60 min point (Figure 5(c)). The t_1/2_, and MRT were 39.6 min and 57.1 min (Table S1). Organ accumulation greatly changed after free ^125^I correction: accumulation in the bladder and the thyroid decreased by 3‐fold, while liver and spleen accumulation increased by 1.5‐fold (Figure 5(d,e)).

**FIGURE 6:**
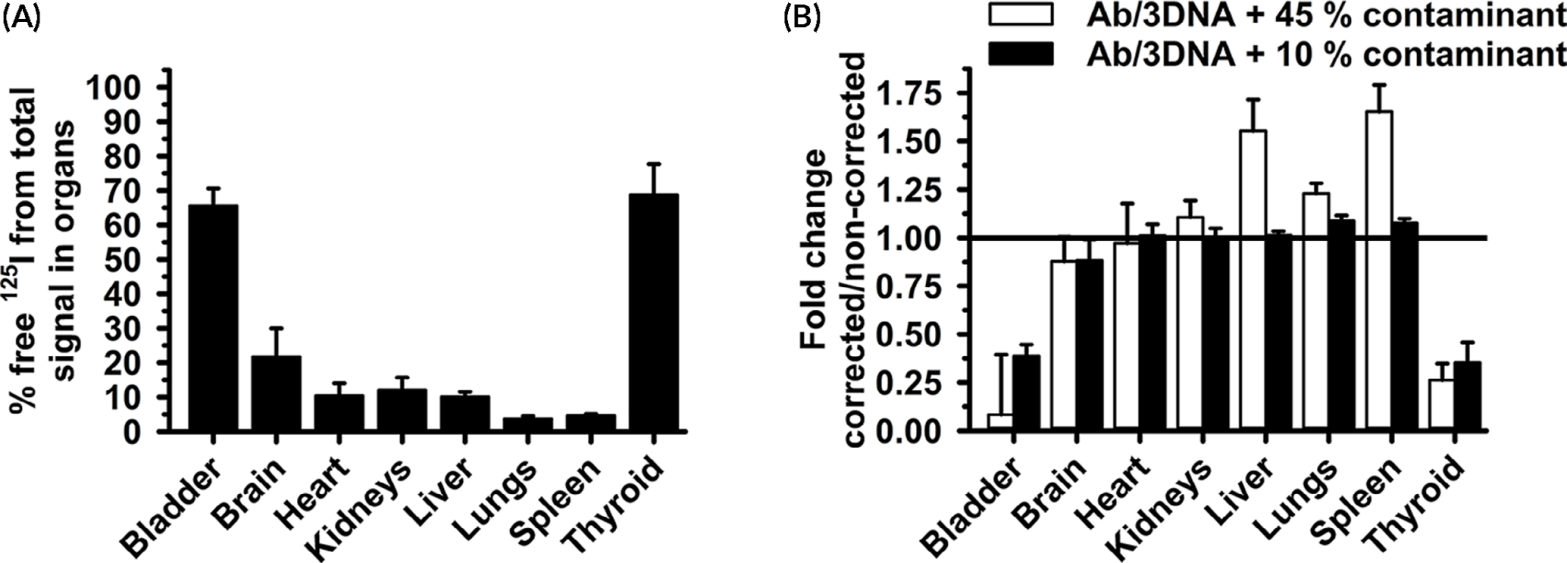
Free ^125^I content and corrected to noncorrected ratio of the biodistribution of ^125^I‐Ab/3DNA containing minimal free ^125^I. (a) ^125^I‐Ab/3DNA containing minimal (10%) free ^125^I was i.v. injected in C57BL/6 mice. Total CPM and free ^125^I CPM were measured prior to and after TCA precipitation, so that the fraction of free ^125^I over the total CPM in each body compartment was calculated. (b) ^125^I‐Ab/3DNA with 10% versus 45% free ^125^I were i.v. injected, and organs were collected and weighed at the indicated times and measured in a γ‐counter prior to and after TCA precipitation to calculate the biodistribution as the percentage of the injected dose (%ID). This was done from noncorrected data (total CPM in tissue/total CPM in the injected dose) or corrected values ([total – free ^125^I CPM in tissue]/[total – free ^125^I CPM in the injected dose]), from where the corrected to non‐corrected ratio was calculated. Data are mean ± S.D. (*n* ≥ 5 mice)

Then, we injected mice with ^125^I‐Ab/3DNA mixed with a low amount of free ^125^I (10% vs. 40–50% in the prior experiment). The contribution of free ^125^I CPM to the total CPM found in each tissue was relatively low (Figure 6(a)) and very reduced for the bladder and thyroid which accumulate the free label compared to the high contribution of free ^125^I CPM of the free ^125^I control injection (Figure 2(e)). The biodistribution of ^125^I‐Ab/3DNA containing low free ^125^I level was determined and compared to ^125^I‐Ab/3DNA with high ^125^I level. For both, corrected and noncorrected data ratio (fold difference) was compared. There was little difference between the corrected and noncorrected biodistribution of the formulation carrying low free ^125^I (y≅1 in Figure 6(b); black bars), unlike the formulation with high level of this radiolabel (y ≠ 1 in Figure 6(b); white bars). Therefore, this method is valid to obtain accurate biodistribution data in the presence of free radiolabel which may arise from metabolism or degradation in the body.

### 2.5 Method implementation using targeted versus nonspecific ^125^I‐Ab/3DNA and comparing different biodistribution times

**FIGURE 7:**
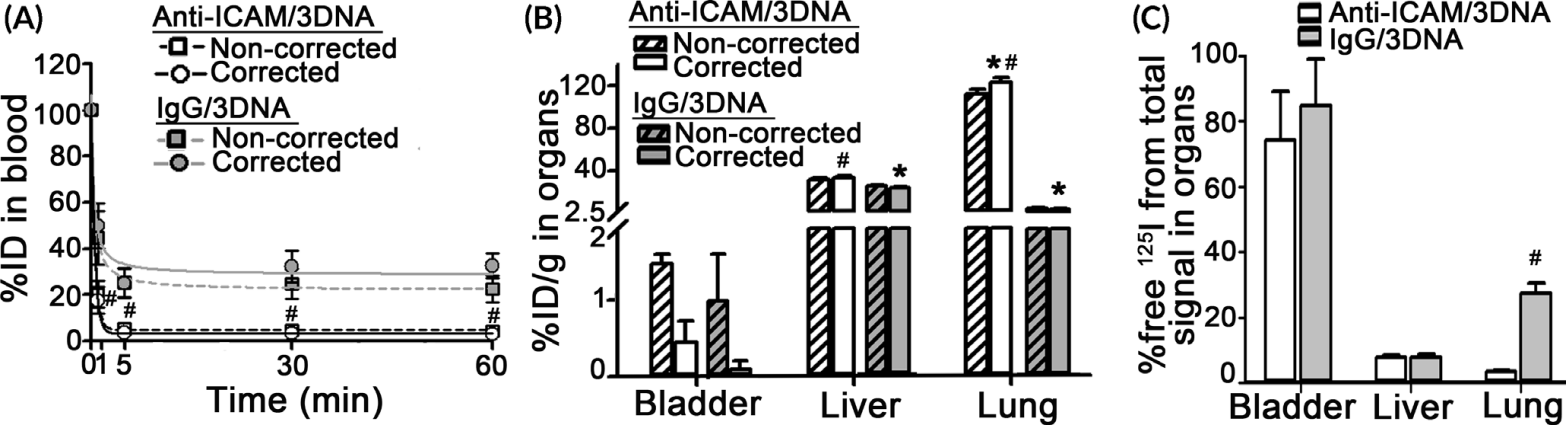
Biodistribution of targeted versus nonspecific 125I‐Ab/3DNA and respective corrections. C57BL/6 mice were injected with ^125^I‐anti‐ICAM/3DNA or nonspecific ^125^I‐IgG/3DNA. (a) Blood samples were collected at the indicated times post‐injection and (b) organs collected at sacrifice at 30 min for CPM measurements in γ‐counter. In both cases, total CPM and free ^125^I CPM were measured in each tissue sample prior to and after TCA precipitation, to implement the empirical correction described in Figure 5. Data are expressed as (a) percentage of the injected dose (%ID) or (b) %ID per gram organ (%ID/g) to show organ concentration, calculated as noncorrected (hashed bars) or corrected (solid bars) to subtract free ^125^I CPM in each tissue. (c) The fraction of free ^125^I over the total CPM in each body compartment was calculated. Data are mean ± S.D. (*n* = 3–5 mice). *Compares corrected to noncorrected data, ^#^Compares IgG/3DNA to anti‐ICAM/3DNA (*p* < 0.05 by Student's *t*‐test)

We next used this method to determine the biodistribution of targeted ^125^I‐Ab/3DNA, using an Ab‐oligo recognizing ICAM‐1 (Figure 7). This is a cell‐surface protein preferentially expressed on the lung endothelium, which provides lung‐specific targeting.^37^ This formulation had 179 ± 6 nm diameter, 0.25 ± 0.017 PDI, and −38 ± 0.6 mV ζ‐potential, similar to IgG/3DNA described above. Both for noncorrected and corrected data, ^125^I‐anti‐ICAM/3DNA disappeared faster from the circulation than the nonspecific formulation (Figure 7(a)). For example, for corrected data, t_1/2_ and MRT were 0.36 min and 0.57 min compared to 39.6 min and 57.1 min for ^125^I‐IgG/3DNA (Table S1). This is known to be due to fast targeting of lung ICAM‐1, confirmed here. For example, corrected data showed 122 %ID/g of lung for the targeted formulation versus 4 %ID/g of lung for the control (Figure 7(b)). Biodistribution in the liver and bladder, examples of metabolic clearance and excretion organs, was much lower (33 and 0.5 %ID/g for anti‐ICAM/3DNA) and closer to IgG/3DNA levels (24 and 0.1 %ID/g for IgG/3DNA). A more specific lung targeting for anti‐ICAM/3DNA over IgG/3DNA was found by using this method: 22‐fold difference using noncorrected data versus 33‐fold difference using corrected data. High free ^125^I found in the lung for IgG/3DNA versus low free ^125^I for anti‐ICAM/3DNA (27 vs. 4% of all lung signal) explains this difference (Figure 7(c)). Free ^125^I found in the liver and bladder was similar for both formulations (e.g., 7.8 and 7.6% of all liver signal), the latter organ carrying much higher levels 85 and 75% of all bladder signal), as expected.

**FIGURE 8:**
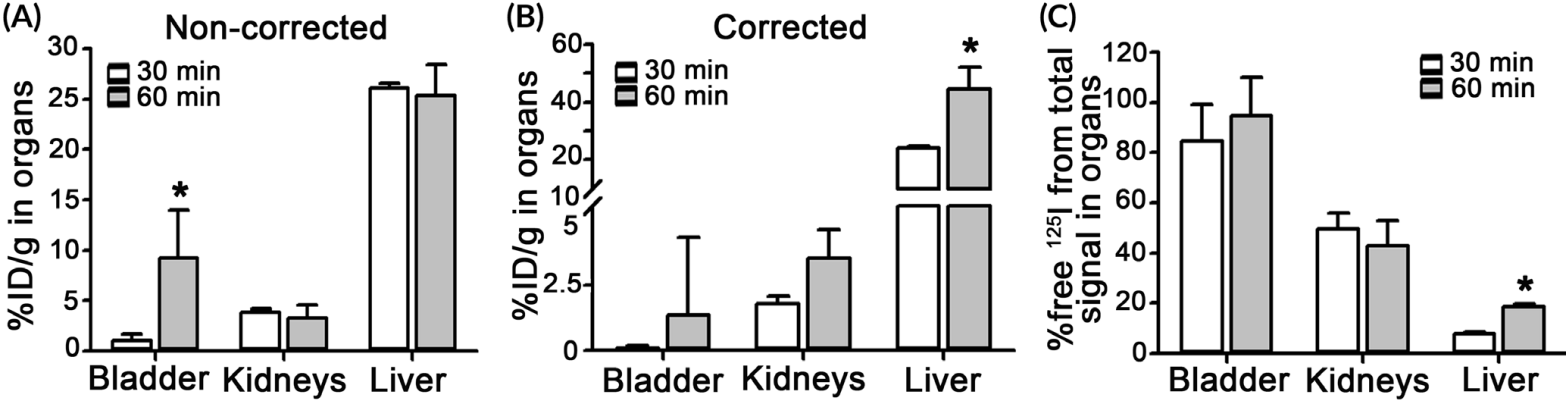
Biodistribution of ^125^I‐Ab/3DNA at different times and respective corrections. C57BL/6 mice were injected with ^125^I‐Ab/3DNA. Organs were collected at sacrifice at 30 min or 60 min post‐injection for γ‐counter measurement of total CPM and free ^125^I CPM prior to and after TCA precipitation, so that the fraction of free ^125^I over the total CPM in each organ was calculated. (a) Data are expressed as %ID per gram organ (%ID/g) to show organ concentration, represented as noncorrected or represented as (b) corrected by subtracting free ^125^I CPM in each tissue. (c) Fraction of free ^125^I over the total CPM in each organ. Data are mean ± S.D. (*n* = 3–5 mice). *Compares 30 min versus 60 min (*p* < 0.05 by Student's *t*‐test)

Finally, using IgG/3DNA, we implemented this method to determine biodistribution changes that may result from time‐dependent degradation in the body (Figure 8). Noncorrected data (Figure 8(a)) showed no change in the kidneys between 30 min and 60 min after injection (3.8 and 3.3 %ID/g), increased bladder levels (1 and 9 %ID/g), and similar liver levels (26 and 25 %ID/g). This is counterintuitive since nanoformulations are large enough to avoid kidney filtration and not expected to be excreted into the bladder, while the liver is expected to collect circulating DDSs over time. This was observed after data correction: from 24 to 44 %ID/g increase in the liver and similar levels (0.1 and 1.5 %ID/g) in the bladder (Figure 8(b)). Also consistent with expectation (Figure 8(c)), free ^125^I increased in the liver (8 to 19%) with time, as expected due to hepatic metabolization.

## 3 DISCUSSION

Radiotracing using γ emitters is considered the gold standard for biodistribution measurements as it provides high accuracy, sensitivity, and throughput abilities.^6^, ^7^ However, as for other labeling methods,^6^ biodistribution data can be confounded by potential tracing of free‐label generated prior to or after administration of biodegradable DDS. Hence, biodistribution studies could benefit from means to determine and correct for these artifacts. In this study, we employed TCA precipitation on blood and tissue samples to quantify the present or generated free radiolabel present or generated and correct biodistribution data accordingly.

We showed that some free ^125^I is released from ^125^I‐Ab/3DNA upon incubation in vitro with buffer, blood or tissues (Figure 1), validating the need for correction methods. Next, using in vivo injection of free ^125^I, we showed each organ accumulated different radiolabel levels (Figure 2). The thyroid accumulated the highest ^125^I concentration, likely due to the sodium‐iodide symporter (NIS).^41^, ^42^ Although kidneys also express NIS,^42^ we did not see high accumulation here, possibly due to ^125^I secretion in the urine. In fact, the bladder accumulated the second greatest free ^125^I concentration (Figure 2(c)). Other organs showed low free ^125^I concentrations (Figure 2(c)), although the absolute accumulation was more prominent in the liver (Figure 2(b)), perhaps due to its fenestrated endothelium and large volume. Hence, the level of free ^125^I widely varies among the different body compartments, justifying the need to correct ^125^I data for free label in each compartment. The implemented method allowed precise classification of all detected ^125^I as a free species in the organs tested (Figure 2(d,e)), indicating that free ^125^I was not incorporated in relevant amounts into body molecules, which would otherwise be precipitated by TCA.

Then, we tested the method using ^125^I‐Ab/3DNA containing a known amount of free ^125^I, which demonstrated the confounding effects of free radiolabel on biodistribution results. We used the biodistribution of free ^125^I from the control experiment to correct for the presence of the free ^125^I in the blood and organs of these animals (Figure 3). This showed an increased accumulation in the liver and spleen and a thyroid decrease, expected for a nanodevice. Unexpectedly, we observed the biodistribution of free ^125^I co‐injected with ^125^I‐Ab/3DNA differed from the biodistribution of free ^125^I administered alone (Figure 4). Speculatively, ^125^I‐Ab/3DNA may compete with free ^125^I for access to certain organs or free ^125^I may arise from ^125^I‐Ab/3DNA degradation. Since the liver plays a role in the degradation, increased free ^125^I in this organ in animals injected with ^125^I‐Ab/3DNA + free ^125^I may be due to some ^125^I‐Ab/3DNA degradation in the liver, as suggested by ex vivo ^125^I release from ^125^I‐Ab/3DNA in liver homogenates (Figure 1). This phenomenon would be less significant in organs unrelated to clearance/degradation, such as the heart. Additionally, as other DDSs,^43^ ^125^I‐Ab/3DNA may form a protein corona containing albumin, which is known to carry ^125^I.^39^ If an albumin corona forms on ^125^I‐Ab/3DNA, some free ^125^I may associate to it in the body. Since ^125^I‐Ab/3DNA accumulates significantly in the liver, it may carry here free ^125^I via protein corona, accounting for the different biodistribution of free ^125^I co‐injected with ^125^I‐Ab/3DNA.

This phenomenon indicates the biodistribution of ^125^I‐Ab/3DNA cannot be corrected using the biodistribution of free ^125^I alone, but free ^125^I must be measured in each organ sample within the same experiment. The comparison of corrected vs. noncorrected biodistribution of ^125^I‐Ab/3DNA showed clear differences in organ biodistribution (Figure 5(d)), there was lower accumulation in organs which predominantly take free radiolabel but are not expected to accumulate ^125^I‐Ab/3DNA (bladder and thyroid; Figure 5(d,e)), removing this confounding signal. Accumulation of ^125^I‐Ab/3DNA in the liver and spleen increased upon correction (Figure 5(d)), in agreement with reports on Ab/nanovehicles of similar size and ζ‐potential (~30–50% ID/g).^19^ In organs where free ^125^I did not substantially accumulate (brain, heart, lungs; Figures 2(b,c) and 4(b,c)), correction did not impact the biodistribution data (Figure 5(d,e)). This correction significantly impacted the comparative biodistribution of targeted versus a nontargeted formulations, anti‐ICAM/3DNA versus IgG/3DNA in this case (Figure 7(b)), for example, from 22‐ to 33‐fold difference, highlighting the relevance of this method. The method also demonstrated value in better determining changes in biodistribution over time (Figure 8(a,b)) and in vivo degradation patterns (Figure 8(c)). This sensitivity is facilitated by the easiness of the implemented method, for example, samples extracted from the animal can be processed at 4°C and measured immediately after collection without the need for complex processing. Inhibitors could be added to the collected samples, for example, azide to stop metabolic activity and inhibit serine proteases, to minimize post‐collection degradation. The differences measured in our study regarding biodistribution of Ab/3DNA and free ^125^I content in organs at different times validate the adequacy of the method for such studies.

## 4 MATERIALS AND METHODS

### 4.1 Reagents and mice

Nonspecific rat immunoglobulin G Ab was from Jackson Immunoresearch (Pike West Grove, PA) and mouse anti‐human ICAM‐1 clone YN.1 was from American Type Culture Collection (Manassas, VA). 72‐mer DNA oligo with 5' thiol modification was from Oligo Factory (Holliston, MA). Pierce Bond‐Breaker TCEP Solution, LC‐SMCC Crosslinker, Zeba Spin Columns (7k MWCO), Thiophilic Adsorption Resin, Amicon 10k MWCO spin filters, and Heterobifunctional Crosslinking Kit were from Fisher Scientific (Kerrville, TX). Iodogen tubes were from Pierce (Rockford, IL) and BioSpin Tris Columns from BioRad (Hercules, CA). Na^125^I was from Perkin‐Elmer (Waltham, MA). All other reagents were from Sigma Chemical (St. Louis, MO). Eight‐week old C57BL/6 mice were from Jackson Laboratory (Bar Harbor, ME). All animal experiments were performed under University of Maryland IACUC approval and adhered to the Principles of Laboratory Animal Care.

### 4.2 Preparation of Ab‐oligonucleotide conjugates and their radiolabeling

We used a 4‐layer 3DNA® (Genisphere LLC) with surface arms coupled to ^125^I‐Ab‐oligo. Ab‐oligo conjugations were performed at Genisphere. 72‐mer DNA oligo with a 5'thiol modification was reduced for 1 h in 50 mM TCEP at 25°C. TCEP was removed by ethanol precipitation and the oligo resuspended in phosphate buffer saline (PBS) + 5 mM EDTA. Ab was reacted with excess LC‐SMCC crosslinker Zeba spin columns, equilibrated in PBS + 5 mM EDTA, were used to remove unreacted LC‐SMCC from the reaction and the reduced oligo was added to Ab‐LC‐SMCC and incubated at 25°C for 12 h. Thiophilic adsorption chromatography was utilized to remove unreacted thiol oligo from the conjugate and conjugate fractions were pooled and concentrated using Amicon 10 kDa MWCO spin filters. The conjugate was radiolabeled using 20 μCi of Na^125^I incubated at 4°C for 5 min in iodination tubes containing 1 μg/μl Ab in PBS. Samples were subjected to size exclusion chromatography in 6 kDa cutoff Tris columns, and centrifuged at 1000*g* for 4 min to eliminate free ^125^I. ^125^I‐Ab‐oligo conjugate was measured in a γ‐counter (2470 Wizard^2^™, Perkin Elmer, Waltham, MA) to estimate counts of detected radioactive events per minute (CPM).

### 4.3 Preparation of 3DNA model nanocarrier and coupling to ^125^I‐Ab‐oligonuleotide

Four‐layer 3DNA was synthesized as published.^33^, ^34^, ^35^ Seven single‐stranded DNA oligonucleotides were hybridized by complementarity into five different “monomer” structures, each with a central double‐stranded region and four single‐stranded ends. Monomers were hybridized to one another up to layer 4, with psoralen crosslinking for stabilization. The resulting scaffold of double‐stranded DNA contained peripheral single‐stranded DNA “arms” for hybridization with ^125^I‐Ab‐oligo,^35^, ^37^ achieved by mixing them at 37°C for 30 min at an appropriate molar ratio (1 μM conjugate, expressed as oligo, to 5.27 μM 4‐layer 3DNA). Size‐exclusion chromatography to separate ^125^I‐Ab/3DNA from uncoupled ^125^I‐Ab‐oligo showed 85–89% coupling success.

### 4.4 Label detachment from ^125^I‐Ab/3DNA in buffer, blood, and organ homogenates

C57BL/6 mice were anesthetized to collect 500 μl blood samples from the retro‐orbital sinus and sacrificed to extract the kidneys and liver, which were homogenized at 28,000 rpm using a Kinematica Polytron™ PT 3100D homogenizer (Kinematica, Lucerne, Switzerland). ^125^I‐Ab/3DNA was added to the blood or organ homogenates, or control PBS‐3% BSA, and incubated at 37 °C. To detect free ^125^I, samples were mixed with 3% BSA in PBS up to 1 ml, then precipitated by adding 200 μl TCA (17% v/v final concentration), incubation for 15 min at 4°C, and centrifugation at 2687*g* for 10 min. Using a γ‐counter, free ^125^I CPM was determined from 600 μl of the sample supernatants and total CPM of the samples, measured prior to TCA precipitation, was used to calculate the percent of free ^125^I released compared to time = 0 from ^125^I‐Ab/3DNA as follows:

$$\%{\text{free}}^{125}I=100\times \frac{2\times \mathrm{CPM}\;\text{supernatant}\;\left(600\;\mathrm{\mu l}\right)}{\mathrm{CPM}\;\text{total}\;\left(1200\;\mathrm{\mu l}\right)}$$

### 4.5 Biodistribution of free ^125^I and ^125^I‐Ab/3DNA in mice

Anesthetized C57BL/6 mice were injected i.v. with ^125^I‐Ab/3DNA + a known amount of free ^125^I or free ^125^I alone. ^125^I was adjusted so that the radioactive signal was between 60 and 1.5× 10^6^ CPM. ^125^I‐Ab/3DNA was injected at 2.15×10^13^ 3DNA particles per kg of body weight and 249 μg Ab per kg of body weight, carrying 30 kCPM free ^125^I. Hence, free ^125^I control was injected at 1.3×10^6^–1.4×10^6^ CPM for a matching dose. Blood samples (100 μl) were collected from the retro‐orbital sinus at selected time points until sacrifice at 30 or 60 min, followed by organ collection. All samples were kept on ice, except during their weighing and radioactivity measurements in a γ‐counter.

Total CPM data were used as obtained to calculate biodistribution parameters (“noncorrected” biodistribution). The known fraction of free ^125^I measured in the original (pre‐injected) formulation or experimentally determined post‐injection in each of the blood and tissue samples, were used to calculate the “corrected” biodistribution. For this, the TCA assay was used with minor modifications: samples were mixed with 3% BSA in PBS up to a 1 ml or 1.7 ml total volume for blood or organ samples. Blood and homogenized organ samples were precipitated by adding 200 μl and 300 μl TCA (17% and 15% v/v final TCA). Precipitation was carried by centrifugation at 2687*g* for either 10 min for blood or 30 min for organ samples. Free ^125^I CPM were determined from 600 μl of the blood sample supernatants or 1 ml of the organ sample supernatants. Total CPM, measured prior to precipitation, were corrected by subtracting the free ^125^I measured after TCA precipitation. Corrected and noncorrected CPM were used to calculate biodistribution parameters, including the percent of the injected dose (%ID) in blood and each organ and the same parameter divided per gram of organ (%ID/g), which reflects a concentration in the blood or tissue sample, as follows:

Non correctedCPM=TotalCPM,

CorrectedCPM=TotalCPM−Free IodineCPM,

$$\%\mathrm{ID}=100\times \frac{\mathrm{CPM}\;\text{organ}\;\left(\text{corrected or non corrected}\;\mathrm{CPM}\right)}{\mathrm{CPM}\;\text{dose}\;\left(\text{corrected or non corrected}\;\mathrm{CPM}\right)},$$

$$\%\mathrm{ID}/g=100\times \frac{\mathrm{CPM}\;\mathrm{per}\;\text{gram organ}\;\left(\text{corrected or non corrected}\;\mathrm{CPM}\right)}{\mathrm{CPM}\;\text{dose}\;\left(\text{corrected or non corrected}\;\mathrm{CPM}\right)}.$$

Pharmacokinetic data such as half‐life (t_1/2_), area under the curve (AUC), clearance, and mean residence time (MRT) were calculated using PKSolver in Microsoft Excel.^44^

### 4.6 Statistical Analysis

Data were calculated as mean ± standard deviation (S.D.). Experiments encompassed *n* ≥ 4 mice. Significance was determined using the Student's unpaired *t*‐test, assuming a *p*‐value of 0.05.

## 5 CONCLUSION

Results indicate the presence of free ^125^I radiolabel in biodistribution samples, which could be generated due to DDS degradation prior to or after administration, needs to be accounted within the same experiment for each organ or blood sample to obtain accurate biodistribution results. This can be achieved by implementing TCA precipitation of said samples, permitting the accurate classification of signals originating from free radiolabel vs. radiolabeled carrier. This method is valuable to characterize DDS parameters such as the presence of free radiolabel in the dose to be administered, interaction of free radiolabel with DDS itself, and in vivo stability or degradation profiles of the formulation, for example, in different organs that may have different capacities to metabolize the labeled DDS components.

## CONFLICT OF INTEREST

Lou Casta, Jessica Bowers, and Robert C. Getts are members of Genisphere LLC.

## AUTHORS CONTRIBUTIONS

Nikša Roki performed and analyzed majority of the experiments, drafted the manuscript, and prepared figures. Melani Solomon analyzed some experiments, prepared figures, and edited the manuscript. Lou Casta characterized Ab‐oligo conjugates. Jessica Bowers and Robert C. Getts provided 3DNA, helped with DLS measurements and data interpretation, and edited the manuscript. Silvia Muro conceptualized and directed the investigation, helped interpret the results, planned the manuscript, and helped write and edit the text and figures.

## Supporting information


**Appendix S1:** Supporting Information

## Data Availability

Authors can make data available upon request
