# Supplementary material for: A method to improve quantitative radiotracing‐based analysis of the in vivo biodistribution of drug carriers
Source: Bioeng Transl Med. 2021 Feb 13;6(2):e10208. doi: 10.1002/btm2.10208 (PMC8126812; doi:10.1002/btm2.10208)
Supplement: Supplementary file 1 — Appendix S1: Supporting Information [file BTM2-6-e10208-s001.docx]

**A Method to Improve Quantitative Radiotracing-Based Analysis of the *In Vivo* Biodistribution of Drug Carriers**

Nikša Roki^1^, Melani Solomon^2^, Lou Casta^3^, Jessica Bowers^3^, Robert C. Getts^3^, and Silvia Muro^2,4,5^*

^1^Fischell Department of Bioengineering, University of Maryland, College Park, Maryland, USA

^2^Institute for Bioscience and Biotechnology Research, University of Maryland, College Park, Maryland, USA

^3^Genisphere, LLC, Hatfield, Pennsylvania, USA

^4^Institute for Bioengineering of Catalonia of the Barcelona Institute of Science and Technology, Barcelona, Spain

^5^Institution of Catalonia for Research and Advanced Studies, Barcelona, Spain

*** Correspondence:**Silvia Muro
[muro@umd.edu](mailto:muro@umd.edu)

Keywords: drug delivery carriers, *in vivo* biodistribution, radiotracing, degradation, free label, trichloroacetic acid precipitation, biodistribution data correction.

**Figure S1.** **Study strategy.** The goal was to validate a method capable of separating free radiotracer (^125^I) which may arise from instability of a radiolabeled drug carrier formulation, in order to correct and obtain more accurate *in vivo* biodistribution data. For this purpose, C57BL/6 mice were intravenously injected with control free ^125^I alone or with a DNA-built nanocarrier, 3DNA®, coupled to ^125^I-labeled antibody-oligonucleotide (Ab-oligo) which contained a known fraction of free ^125^I contaminant. Blood and organs were extracted either 30 min or 1 h after injection, homogenized, and precipitated with trichloroacetic acid (TCA) to determine total and free ^125^I CPM, which provided correction for more accurate *in vivo* biodistribution data.

**Supplementary Table S1.** Blood pharmacokinetic parameters

| **Condition** | **t_1/2_**  **(min)** | **CL (%ID/min)** | **AUC (%ID**$\boldsymbol{\times}$**min)** | **MRT**  **(min)** |
| --- | --- | --- | --- | --- |
| **Fig. 2a – Control free ^125^I** | 1.30 | 0.56 | 178.29 | 1.87 |
| **Fig. 3a – ^125^I-Ab/3DNA – Non-corrected** | 20.32 | 0.05 | 1613.40 | 29.31 |
| **Fig. 3a – ^125^I-Ab/3DNA – Corrected using control free ^125^I from Fig. 2a** | 48.12 | 0.03 | 2215.94 | 69.42 |
| **Fig. 4a – Free ^125^I contained in ^125^I-Ab/3DNA formulation** | 1.51 | 0.49 | 203.57 | 2.17 |
| **Fig. 5c – ^125^I-Ab/3DNA - Corrected using free ^125^I in the formulation, from Fig. 4a** | 39.56 | 0.03 | 2293.74 | 57.07 |
| **Fig. 7a – ^125^I-Anti-ICAM/3DNA – Non-corrected** | 0.37 | 0.07 | 1292.77 | 0.62 |
| **Fig. 7a – ^125^I-Anti-ICAM/3DNA – Corrected using free ^125^I in the formulation** | 0.36 | 0.09 | 1088.24 | 0.57 |

AUC = area under the curve; CL = clearance; MRT = mean residence time; t_1/2_ = half-life. (n≥5 mice).
